# Bone-added periodontal plastic surgery: a new approach in esthetic dentistry

**DOI:** 10.1186/s13022-015-0010-5

**Published:** 2015-02-11

**Authors:** Gholam Ali Gholami, Hadi Gholami, Reza Amid, Mahdi Kadkhodazadeh, Amir Reza Mehdizadeh, Navid Youssefi

**Affiliations:** Department of Periodontics, Dental School, Shahid Beheshti University of Medical Sciences, Evin, Tehran, Iran; Department of Prosthodontics, Faculty of Dental Medicine, University of Bern, Bern, Switzerland; Dental Research Center, Shahid Beheshti University of Medical Sciences, Evin, Tehran, Iran

**Keywords:** Plastic surgery, Root coverage, Periodontal regeneration, Bone graft

## Abstract

This article proposes a combined technique including bone grafting, connective tissue graft, and coronally advanced flap to create some space for simultaneous bone regrowth and root coverage.

A 23 year-old female was referred to our private clinic with a severe class II Miller recession and lack of attached gingiva. The suggested treatment plan comprised of root coverage combined with xenograft bone particles.

The grafted area healed well and full coverage was achieved at 12-month follow-up visit. Bone-added periodontal plastic surgery can be considered as a practical procedure for management of deep gingival recession without buccal bone plate.

## Introduction

Marginal tissue recession is a mucogingival problem that is considered a major challenge for clinicians and patients. It is frequentlyassociated with esthetic concerns, fear of tooth loss, root caries, and dentin hypersensitivity. Manyprotocols are available for the management ofsuch defects including different types of soft tissue grafts. Several studies have confirmed thatthese recessions can be predictably covered by various surgical procedures like as pedicle flaps, subepithelial connective tissue grafts (CTG) with or without coronally positioned flap (CPF), and guided tissue regeneration (GTR), if the interdental papilla is not affected [1-5].

In spite of predictable clinical outcomes by using CTGs, its healingprocess and histological outcome still remain controversial. Evidence data of human histology after the use of these techniques are scarce. The histologic evidenceshave been mostly derived from animal studies or some case reportsconducted by the extraction of the treated teeth. It seems that CPF and CTG are associated with somedegrees of periodontal regeneration [6-8]. However, some authorshave reported that healing occurs primarily by a long junctional epithelium or to a limited extent by connective tissueadhesion of the graft [9,10]. The concern about the nature of the grafted tissue attachment is based on the concept that the ultimate goal of periodontal treatment is to fully restore the attachment apparatus. Current available therapies have shown limited and rather unpredictable results. The nature of connective tissue attachment seems to be stable over time, although, ultimate goal of a root coverage procedure should be new bone formation overthe denuded roots.

To our knowledge, there is no report of bone-added periodontal plastic surgery for root coverage procedures in humans. The aim of the present investigation was to present a technique with a combination of bone substitute, CTG, and CPF thatwas used to create some space for osteoconduction and soft tissue coverage over denuded roots.

## Case presentation

**Figure 1:**
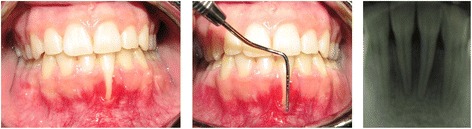
**A severe deep class II Miller gingival recessions in anterior mandibular tooth.**

A 23 year-old female was referred to our private clinic with a chief complaint of hypersensitivity, fear of tooth loss and gingival recession in the mandibular anterior tooth. She was in good general health and non smoker. Intraoral examination showed a good oral hygiene status with a full-moth plaque score equal to 17% [11]. A deep class II Miller recession with the lack of attached gingiva, and narrow band of keratinized tissue was observed (Figure 1). Probing depths (PD) and clinical attachment level (CAL) measurements and registrations of marginal gingival recession (MGR) were obtained using a periodontal probe (UNC 15, Hu-Friedy Mfg. Inc, Chicago, IL, USA). The measurements were rounded up to the nearest millimeter. Tooth mobility was assessed and graded 0-2 and tooth hypersensitivity calculated via visual analysis scale (VAS). Measurements were done by an examiner with more than 10 years of clinical experience. Bone mapping revealed that it was complete lack of buccal bone plate over involved tooth. The suggested treatment plan comprised root coverage combined with xenograft bone particles. Initial therapies, including supra-gingival plaque removal, polishing, occlusal adjustment, and oral hygiene instruction with proper tooth brushing method were performed. Surgical procedures were performed by one of the authors who was not involved with clinical measurements. The clinical measurements were done by other experienced and calibrated investigators who were not informed about the surgical method. In case of controversy in the measurements by the examiners, they were asked to repeat the evaluation to reach a consensus. Another experienced and blinded operator was responsible for the radiographic examination. Written informed consent was obtained from the patient for publication of this case report and any accompanying images. A copy of the written consent is available for review by the Editor-in-Chief of this journal.

### Surgical procedure

**Figure 2:**
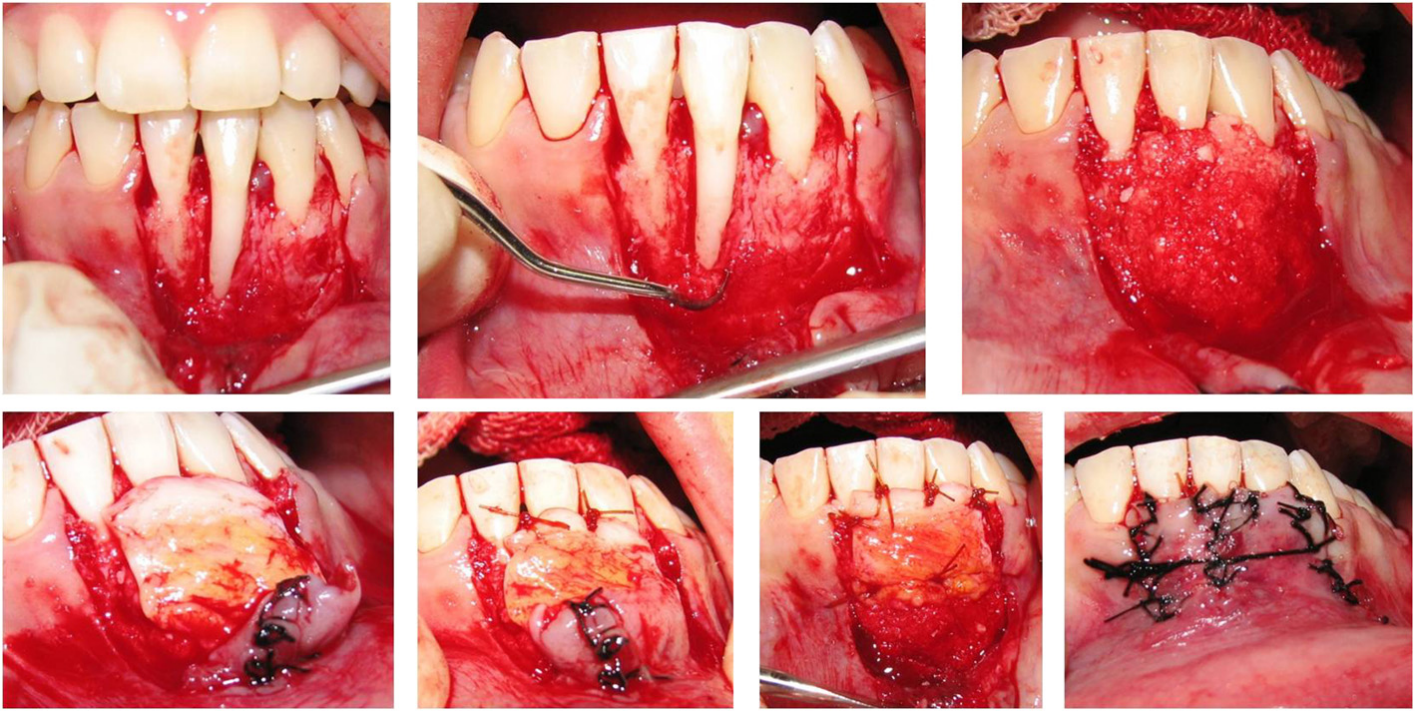
**Surgical procedure: bed preparation, root surface preparation, covering bone dehiscence with xenograft bone particles mixed with blood, stabilization of connective tissue graft over bone graft, double papilla and coronally positioned flap.**

The design of CPF was similar to that described previously by Langer and Langer [12]. After local anesthesia of the recipient site using 2% articainewithepinephrine1:100 000 (Septanest®, Septodont, Spain), an intra crevicular incision was made from right to left mandibular canines. Two verticalreleasing incisions were made along neighboring teeth. A partial thickness flap was elevated with a No.15c surgical blade beyond the mucogingival junction. Thus, it was extended until it could be passively positioned coronally over the defect without tension (Figure 2). The exposedroot surface was debrided completely with a curette (3/4 Gracey curette, Hu-Friedy Mfg. Inc, Chicago, IL, USA) and conditioned with tetracycline powder. Pedicle flaps were sutured to make a single flap. The connective tissue was harvested from palate by trap door technique. In the palate, the distance between the horizontal incision and gingival margin had to be more than 2 mm. By using no.15 scalpel, the epithelium was elevated and then a 1.5- 2.0 mm thickness connective tissue graft was obtained [13]. The epithelium was laid back and sutured with sutures (Silk 4-0, SUPA Co, Tehran, Iran).

Dehiscence bone defects over exposed root surfaces were overfilled with a mixture of deproteinized bovine bone (Bio-Oss Collagen, Geistlich AG, Wolhusen, Switzerland) and blood obtained froma peripheral vein. CTG was trimmed and sutured over the defect with a 5–0 bio-absorbable suture (Vicryl®, ETHICON, Johnson & Johnson, Livingston, Scotland) at the level of CEJs. Previously reflectedpartial-thickness flap was sutured as double papillary technique described by Harris [14], and then coronally positioned to cover the entire graft without tension [15]. The flap was sutured in place with sling sutures (Figure 2).

The patient was instructed to discontinue tooth brushing for 3 weeks and to avoidtrauma at the surgical site. A 0.2% Chlorhexidine digluconate mouthwash (CHX mouthwash, Daroupakhsh Co, Iran) was prescribed twice daily. Professional cleaning was done by a hygienist every day until the sutures were removedon day14. The patients were recalled for prophylaxisat every month, postoperatively. The clinical measurements were recorded at baseline and every 3 months till the final recall visit at six years.

### Clinical outcomes

**Figure 3:**
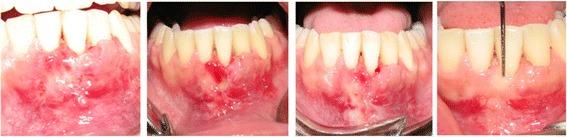
**Post operative follow up showed complete root coverage and significant gain of keratinized tissue.** Photographs were taken 2, 4, 8, and 12 weeks (left to right) after surgical intervention.

**Figure 4:**
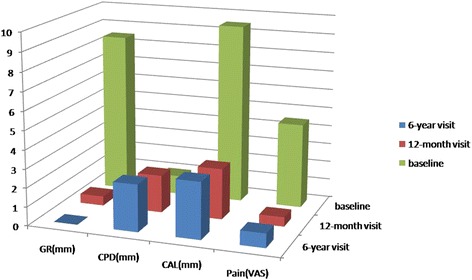
**Clinical improvement of different clinical parameters at baseline, 1- and six-year follow up.**

Wound healing was uneventful. The grafted area healed well and complete coverage was achieved at 12-monthfollow-up visit (Figure 3). Complete root coverage was achievedwith a minimum of 6 mm gain in keratinized tissue. There was also no change in probing depthcompared to the valuebefore thesurgery (Figure 4). The general color and volume match were satisfactory. The clinical and patient-centered outcomes were excellent. No scars resulting in esthetically displeasingappearance were observed. Clinically, the grafted tissues seemed to be attached to the root surfaces.

**Figure 5:**
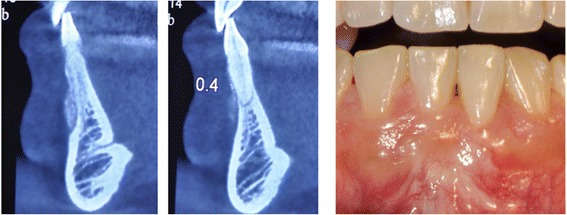
**Remnants of xenograft bone particles over denuded root (left) in comparison to intact neighboringsite(right) at six-year follow up.** Clinical view represents full coverage of the defect.

After six years of regular follow up, patient needed to a 3D imaging for implantation in posterior sites. Cross sectional views that were obtained revealed a clear maintained space over denuded roots which were covered with CTG in clinic. The remnant particles of xenograft were still present on the root surfaces (Figure 5).

## Discussion

Subepithelial connective tissue grafts may be still considered the gold standard procedure for covering Miller Class I and II gingivalrecessions [4]. One of the advantages of CG with a CAF over other procedures is that it produces a larger increase in the keratinized tissue compared to the repositioned flaps alone [14,16]. The presence of thick attached keratinized tissue may act as a protective factor against marginal inflammation or trauma.

Rosetti et al. [17] showed that both SCTG and GTR with a bioabsorbable membrane and bone graft demonstrated significant clinical and esthetic improvement in gingival recession coverage 18 months after surgery. The two procedures were statistically similar in root coverage (SCTG = 95.6%, GTR = 84.2%). In addition, they mentioned that the gingival recessions treated with the SCTG weresuperior in terms of GR, RC, and KT clinical parameters, while GTR demonstrated better PD reduction. The final esthetic results were similar using both techniques. However, occurrence of true regeneration after such procedures has been a controversial issue. McGuire et al. [18] showed that no histological evidence of cementum, bone, or periodontal ligament (PDL) and, therefore, regeneration could be determined using CTG + CPF. Thus, they recommended adding some regenerative materials to enhance the capacity of tissue regeneration.

There are some case reports that used different materials which were combined with CTG to achieve some regeneration over denuded root surfaces. For example, Maurer and Leone [19] used CTG coupled with enamel matrix derivative (Emdogain) to maximize the regenerative potential. Nozawa et al. [20] also described a case of gingival recession in which root coverage and coronal bone regrowth were achieved after treatment with a connective tissue-bone graft and enamel matrix derivative.

It can be concluded that connective tissue grafts are used successfully in periodontal therapy for root coverage. However, reports on the histologic interface between the root surface and the grafted tissue have been few in number [21-25]. Some studies have shown that the pedicle graft withor without CTG may heal by periodontal regeneration.Newconnective tissue attachment (3.9 mm), including periodontalligament was observed to be associated with aCAF with CTG [6]. Histological evaluation of the CGrevealed a healing process characterized by one mm newbone formation, new periodontal fibers, new cementum, and new connective tissue attachment [7].

CTG and CAF plus Emdogain® (Enamel Matrix Derivative)were associated with periodontal regeneration(1.87 mm of new bone, and 2.25 mm of connective tissue anchored in 0.06 mm of new cementum) [8]. However, the results of the study done by Carnio et al. suggest that a combination of CT grafts and EMD results mainly in an adhesion between the CT and root surface [23]. Some degrees of periodontal regeneration may occur in some regions. A CG under aCAF was associated with partial root coverage and along junctional epithelium, with a minimal new attachmentand bone formation [21]. Regeneration is also possiblein other periodontal plastic surgery procedures such as free gingival graft, Acellular Dermal MatrixGraft and guided tissue regeneration [3]. Few investigations can be found in the literature on the histological nature of the attachment of connective tissue grafts to root surfaces previously exposed by recession (Table 1).

**Table 1:** **Summary of published data with histologic findings of different root coverage procedures**

**Author/year**	**Materials/methods**	**Results (histologic)**
**Pasquinelli/1995** [22]	FGG+ tetracycline conditioning	4.4 mm of new attachment and 4.0 mm of new bone growth.
**Harris/1999** [9]	CTG+ Partial DPF	Two different healing patterns: first onewas a long junctional epithelial attachment with minimal connective tissue
		The other pattern was a short junctional epithelium and predominately connective tissue.
		No new bone or cementum was seen in any section.
**Goldstein et al/2001** [6]	Periosteal CTG	Sulcular epithelium was keratinized; epithelium lining the dentin exhibited rete ridges projecting into the gingival connective tissue; and junctional epithelium extended over new cementum. New connective tissue attachment was also observed, including periodontal ligament.
**Majzoub et al/2001** [21]	CTG	Long junctional epithelium throughout the major portion. Only minimal signs of new cementum-like tissue
**Carnio et al/2002** [23]	CTG + EMD	Short junctional epithelium, dense CT fibers were found in close proximity to the root surface,
		No insertion of fibers into the root was observed.
		Newcementum and new bone in the most apical end of the grafted area.
**McGuire et al/2003** [10]	CTG	A connective tissue attachment
		No histological evidence of cementum, bone, or PDL.
**Cummings et al/2005** [24]	CTG	Cementum deposition within the root notches, unaffected alveolar bone.
**McGuire et al/2009** [18]	CTG + rhPDGF + beta-TCP	Evidence of regeneration of cementum, PDL with inserting connective tissue fibers, and supporting alveolar bone, none of the CTG-treated sites exhibited any signs of periodontal regeneration.
**Roman et al/2010** [25]	CTG	No ligament or bone, no sign of a long junctional epithelium a long connective tissue attachment

More than 9mm root coverage which was detected in this case report could be achieved through following clinical guidelines:

- a- Preparation of an extensive bed for sufficient blood supply,
- b- Using xenograft bone particles over denuded root surfaces for space maintaining,
- c- Adding own patients blood mixed with bone graft granules for accelerating the healing during initial phase,
- d- Excellent stabilization od connective tissue graft over bone particles at the level of CEJs,
- e- Using double pedicle and coronally positioned flaps for covering the connective tissue graft completely, and
- f- Strict follow up protocol with optimal level of patient’s cooperation.

As shown in Figure 5, long term stability of xenograft particles was clearly shown in a cross sectional view of the treated sites via a cone beam computed tomography (CBCT) obtained 6 years later. Although this imaging method cannotbe used to indicate a true regeneration, the differences between treated and untreated sites revealed that it is more reasonable to use bone graft to cover the denuded root surfaces.

## Conclusions

Bone added periodontal plastic surgery can be considered as a safe, efficient, and stable technique for complete root coverage ofsevere deep class II gingival recessions combined with lack of buccal plates. Clinical trials with sufficient cases are necessary to compare the results withthose of more conventional procedures.

## References

[1] Roccuzzo M, Bunino M, Needleman I, Sanz M (2002). Periodontal plastic surgery for treatment of localized gingival recessions: a systematic review. J Clin Periodontol.

[2] Oates TW, Robinson M, Gunsolley JC (2003). Surgical therapies for the treatment of gingival recession. A systematic review. Ann Periodontol.

[3] Sedon CL, Breult LG, Covington LL, Bishop BG (2005). The subepithelial connective tissue graft: Part II. Histologic healing and clinical root coverage. J Contemp Dent Pract.

[4] Chambrone L, Chambrone D, Pustiglioni FE, Chambrone LA, Lima LA (2008). Can subepithelial connective tissue grafts be considered the gold standard procedure in the treatment of Miller Class I and II recession-type defects?. J Dent.

[5] Chambrone L, Sukekava F, Araujo MG, Pustiglioni FE, Chambrone LA, Lima LA (2009). Root-coverage procedures for the treatment of localized recession-type defects. Cochrane Database Syst Rev.

[6] Goldstein M, Boyan BD, Cochran DL, Schwartz Z (2001). Human histology of a new attachment after root coverage using subepithelial connective tissue graft. J Clin Periodontol.

[7] Paolantonio M (2002). Treatment of gingival recessions by combined periodontal regenerative technique, guided tissue regeneration, and subpedicle connective tissue graft. A comparative clinical study. J Periodontol.

[8] Rasperini G, Silvestri M, Schenk RK, Nevins ML (2000). Clinical and histologic evaluation of human gingival recession treated with a subepithelial connective tissue graft and enamel matrix derivative (Emdogain): a case report. Int J Periodontics Restorative Dent.

[9] Harris RJ (1999). Human histologic evaluation of root coverage obtained with a connective tissue with partial thickness double pedicle graft. A case report. J Periodontol.

[10] McGuire MK, Cochran DL (2003). Evaluation of human recession defects treated with coronally advanced flaps and either enamel matrix derivative or connective tissue. Part 2: Histological evaluation. J Periodontol.

[11] O’leary TJ, Drake RB, Nayor JE (1972). The plaque control record. J Periodontol.

[12] Langer B, Langer L (1985). Subepithelial connective tissue graft technique for root coverage. J Periodontol.

[13] Nemcovsky CE, Artzi Z, Tal H, Kozlovsky A, Moses O (2004). A multicenter comparative study of two root coverage procedures: coronally advanced flap with addition of enamel matrix proteins and subpedicle connective tissue graft. J Periodontol.

[14] Harris RJ (2002). Connective tissue grafts combined with either double pedicle grafts or coronally positioned pedicle grafts: results of 266 consecutively treated defects in 200 patients. Int J Periodontics Restorative Dent.

[15] Greenstein G, Greenstein B, Cavallaro J, Elian N, Tarnow D (2009). Flap advancement: practical techniques to attain tension-free primary closure. J Periodontol.

[16] Sugarman EF (1969). A clinical and histological study of the attachment of grafted tissue to bone and teeth. J Periodontol.

[17] Rosetti EP, Marcantonio RA, Rossa C, Chaves ES, Goissis G, Marcantonio E (2000). Treatment of gingival recession: comparative study between subepithelial connective tissue graft and guided tissue regeneration. J Periodontol.

[18] McGuire MK, Scheyer ET, Schupbach P (2009). Growth factor-mediated treatment of recession defects: a randomized controlled trial and histologic and microcomputed tomography examination. J Periodontol.

[19] Maurer S, Leone CW (2001). Use of a serially layered, double connective tissue graft approach to enhance maxillary anterior esthetics. Int J Periodontics Restorative Dent.

[20] Nozawa T, Sugiyama T, Satoh T, Tanaka K, Enomoto H, Ito K (2002). Connective tissue-bone onlay graft with enamel matrix derivative for treatment of gingival recession: a case report. Int J Periodontics Restorative Dent.

[21] Majzoub Z, Landi L, Grusovin MG, Cordioli G (2001). Histology of connective tissue graft. A case report. J Periodontol.

[22] Pasquinelli KL (1995). The histology of new attachment utilizing a thick autogenous soft tissue graft in an area of deep recession: a case report. Int J Periodontics Restorative Dent.

[23] Carnio J, Camargo PM, Kenney EB, Schenk RK (2002). Histological evaluation of 4 cases of root coverage following a connective tissue graft combined with an enamel matrix derivative preparation. J Periodontol.

[24] Cummings LC, Kaldahl WB, Allen EP (2005). Histologic evaluation of autogenous connective tissue and acellular dermal matrix grafts in humans. J Periodontol.

[25] Roman A, Câmpian R, Domşa I, Soancă A, Gocan H (2010). Subepithelial connective tissue graft for root coverage: clinical case reports and histologic evaluation. Rom J Morphol Embryol.

